# Combined intervention with pioglitazone and *n*-3 fatty acids in metformin-treated type 2 diabetic patients: improvement of lipid metabolism

**DOI:** 10.1186/s12986-015-0047-9

**Published:** 2015-12-02

**Authors:** Jiri Veleba, Jan Kopecky, Petra Janovska, Ondrej Kuda, Olga Horakova, Hana Malinska, Ludmila Kazdova, Olena Oliyarnyk, Vojtech Skop, Jaroslava Trnovska, Milan Hajek, Antonin Skoch, Pavel Flachs, Kristina Bardova, Martin Rossmeisl, Josune Olza, Gabriela Salim de Castro, Philip C. Calder, Alzbeta Gardlo, Eva Fiserova, Jørgen Jensen, Morten Bryhn, Jan Kopecky, Terezie Pelikanova

**Affiliations:** Institute for Clinical and Experimental Medicine, Prague, Czech Republic; Department of Adipose Tissue Biology, Institute of Physiology of the Czech Academy of Sciences, Prague, Czech Republic; Human Development & Health Academic Unit, Faculty of Medicine, University of Southampton, Southampton, UK; Department of Mathematical Analysis and Applications of Mathematics, Faculty of Science, Palacky University, Olomouc, Czech Republic; Department of Physical Performance, Norwegian School of Sport Sciences, Oslo, Norway; Silentia AS, Svelvik, Norway

**Keywords:** Eicosapentaenoic acid, Docosahexaenoic acid, Indirect calorimetry, Meal test, Humans, Hyperinsulinemic-euglycemic clamp

## Abstract

**Background:**

The marine *n*-3 fatty acids, eicosapentaenoic acid (EPA) and docosahexaenoic acid (DHA) exert numerous beneficial effects on health, but their potency to improve treatment of type 2 diabetic (T2D) patients remains poorly characterized. We aimed to evaluate the effect of a combination intervention using EPA + DHA and the insulin-sensitizing drug pioglitazone in overweight/obese T2D patients already treated with metformin.

**Methods:**

In a parallel-group, four-arm, randomized trial, 69 patients (66 % men) were assigned to 24-week-intervention using: (i) corn oil (5 g/day; Placebo), (ii) pioglitazone (15 mg/day; Pio), (iii) EPA + DHA concentrate (5 g/day, containing ~2.8 g EPA + DHA; Omega-3), or (iv) pioglitazone and EPA + DHA concentrate (Pio& Omega-3). Data from 60 patients were used for the final evaluation. At baseline and after intervention, various metabolic markers, adiponectin and cytokines were evaluated in serum using standard procedures, EPA + DHA content in serum phospholipids was evaluated using shotgun lipidomics and mass spectrometry, and hyperinsulinemic-euglycemic clamp and meal test were also performed. Indirect calorimetry was conducted after the intervention. Primary endpoints were changes from baseline in insulin sensitivity evaluated using hyperinsulinemic-euglycemic clamp and in serum triacylglycerol concentrations in fasting state. Secondary endpoints included changes in fasting glycemia and glycated hemoglobin (HbA_1c_), changes in postprandial glucose, free fatty acid and triacylglycerol concentrations, metabolic flexibility assessed by indirect calorimetry, and inflammatory markers.

**Results:**

Omega-3 and Pio& Omega-3 increased EPA + DHA content in serum phospholipids. Pio and Pio& Omega-3 increased body weight and adiponectin levels. Both fasting glycemia and HbA_1c_ were increased by Omega-3, but were unchanged by Pio& Omega-3. Insulin sensitivity was not affected by Omega-3, while it was improved by Pio& Omega-3. Fasting triacylglycerol concentrations and inflammatory markers were not significantly affected by any of the interventions. Lipid metabolism in the meal test and metabolic flexibility were additively improved by Pio& Omega-3.

**Conclusion:**

Besides preventing a modest negative effect of *n*-3 fatty acids on glycemic control, the combination of pioglitazone and EPA + DHA can be used to improve lipid metabolism in T2D patients on stable metformin therapy.

**Trial registration:**

EudraCT number 2009-011106-42.

**Electronic supplementary material:**

The online version of this article (doi:10.1186/s12986-015-0047-9) contains supplementary material, which is available to authorized users.

## Background

The complex etiology of type 2 diabetes (T2D) prompts for the use of a combination therapy to target multiple underlying mechanisms. Indeed, standards of medical care in diabetes recommend the combination therapy of metformin and other anti-diabetic drugs, next to metformin monotherapy [1]. Major effects of metformin include the lowering of hepatic glucose production and fasting glycemia and reduced risk of cardiovascular events [2], as well as an independent anti-inflammatory action [3] and amelioration of the oxidative stress [4]. The combination of metformin and the peroxisome proliferator-activated receptor (PPAR) γ agonist pioglitazone, a drug from the thiazolidinedione family, provided superior clinical outcomes to metformin alone [5]. In spite of the beneficial effects of thiazolidinediones on glycemic control, insulin sensitivity, inflammation and oxidative stress [2, 4, 5], as well as their triacylglycerol-lowering effect in both humans [5] and mice [6, 7], clinical use of pioglitazone has declined recently due to the risk of its side-effects (reviewed in [1, 8]). However, this risk could be outweighed by the benefits of pioglitazone in prevention of cardiovascular disease [9], the leading cause of death in patients with T2D [10]. Indeed, pioglitazone is associated with a relatively low risk of all-cause mortality (reviewed in [8]).

Dietary interventions represent an important part of any management or treatment strategy for patients with T2D. Naturally occurring long-chain *n*-3 polyunsaturated fatty acids, namely eicosapentaenoic acid (EPA; 20:5 *n*-3) and docosahexaenoic acid (DHA; 22:6 *n*-3), are considered to be healthy dietary constituents in diabetics [11]. These fatty acids exert anti-inflammatory and hypolipidemic effects, while increasing catabolism of lipids *via* a PPARα-mediated mechanism [11, 12]. At a daily dose of 4 g, EPA + DHA are approved for the treatment of hypertriacylglycerolemia [13] and could ameliorate non-alcoholic fatty liver disease [14]. Even modest consumption of EPA + DHA (0.25–0.5 g/day) helps to prevent cardiovascular disease [15], reflecting probably the stabilization of atherosclerotic plaques [16]. The hypolipidemic effect of *n*-3 fatty acids could also be involved, since increased postprandial triacylglycerolemia represents an independent risk factor for cardiovascular disease in T2D patients [10, 17]. In contrast with the earlier clinical trials, most of the recent studies did not show a benefit of EPA + DHA in the secondary prevention of cardiovascular disease (reviewed in [11]). Importantly, in patients with T2D within a large cohort, positive cardiovascular effects of *n*-3 fatty acids were observed [18]. Regarding the effects of EPA + DHA on glycemic control and insulin sensitivity, positive results have been obtained in animal models (reviewed in [11]), and in the prevention of T2D in obese children and young overweight/obese individuals [19]. Mixed results were obtained with respect to prevention of T2D by EPA + DHA in adult humans; in patients with T2D, either no or detrimental effects on glucose homeostasis were found in older studies, while more recent studies mostly showed neutral effects [11].

Our experiments in mice with diet-induced obesity [6, 7, 20] demonstrated additive beneficial effects of a combined intervention using EPA + DHA and thiazolidinediones on insulin sensitivity, glucose tolerance, metabolic flexibility, lipid metabolism, hepatic steatosis, inflammation and obesity. These effects were observed in both the prevention [6, 7, 20] and reversal [7] of obesity-associated phenotypes. Therefore, in this study, we sought to examine whether EPA + DHA, at a dose of ~2.8 g/day (i.e., 5 g EPA + DHA concentrate/day), could modulate the effects of pioglitazone in overweight and obese patients with T2D, specifically in well-controlled patients treated with metformin. In order to unmask potential additive effects, pioglitazone was used at a relatively low dose of 15 mg/day, which is the initial recommended dose for treatment. The major goal of the study was to characterize the effect of the combined intervention on glucose homeostasis and lipid metabolism.

## Materials and methods

### Study design and patients

A 24-week, parallel-group, four-arm, randomized trial (EudraCT number 2009-011106-42) was conducted in accordance with the principles of the Declaration of Helsinki (2008 revision) and with approval by the Institutional Ethical Committee. All patients provided written informed consent prior to their participation.

Inclusion criteria were 40–70 years of age, diagnosis of T2D as defined by the criteria of the American Diabetes Association and recognized by WHO, Expert Committee on the Diagnosis and Classification of Diabetes Mellitus (American Diabetes Association, 2004) diagnosed at least 3 months preceding screening visit, treatment by oral metformin as a monotherapy at a stable dose (0.5–3.0 g/day) for at least 1 month and no other antidiabetic agent, hemoglobin A_1c_ (HbA_1c_) < 80 mmol/mol, fasting plasma triacylglycerols ≤ 6 mmol/l, BMI 25–45 kg/m^2^, ability and willingness to adhere to the protocol and signed and dated written Informed consent obtained before any trial-related activities. Exclusion criteria were type 1 diabetes, uncorrected thyroid dysfunction, significant weight gain or loss (>5 % of total body weight within the past 3 months), therapy with insulin, or warfarin or fibrates within past 3 months (statins and salicylic acid were allowed; 51 % of patients were treated with either simvastatin or atorvastatin), tachycardia (>100 beats/min; or use of stable doses of antihypertensives shorter than 3 months prior the screening and during the trial), history of cardiovascular disease (myocardial infarction in the last year, coronary revascularization including percutaneous transluminal coronary angioplasty, coronary artery bypass graft surgery in the previous year and no subsequent angina, unstable angina, congestive heart failure), pregnancy or lactation, significant renal impairment (serum creatinine >150 μmol/l), chronic or advanced hepato-biliary diseases, history of alcohol or substance abuse within the past year, allergy to any of the capsule excipients, participation in any other clinical trial during the previous 3 months, and clinically significant anemia (hemoglobin < 120 g/l for males and < 110 g/l for females) or any other abnormal hemoglobin profile.

### Procedures

**Fig. 1:**
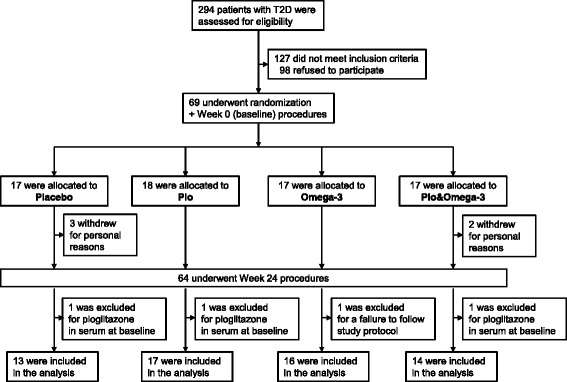
Study design

Out of 294 patients subjected to an initial screening, 69 eligible patients (66 % men) were enrolled (Fig. 1) at the Diabetes Centre, Institute for Clinical and Experimental Medicine, Prague, Czech Republic. All measurements, procedures and sample collection were performed at week 0 and week 24 (2 visits during each week), on an outpatient basis, after overnight (8–10 h) fasting with water *ad libitum.* During week 0 (baseline; 3 weeks after the screening visit maximum), at the first visit, serum and muscle samples were collected (see below and Additional file 1), and a hyperinsulinemic-euglycemic clamp was performed (see below). At the second visit one week later, a standard meal test was performed (see below), followed by proton magnetic resonance spectroscopy (Magnetom Trio, Siemens, Erlangen, Germany) of liver and skeletal muscle to measure lipid content as described in Additional file 1. At the second visit, patients were randomized to (i) 5 g/day corn oil (Placebo), (ii) 15 mg/day pioglitazone (Pio; Actos, Takeda), (iii) 5 g/day EPA + DHA concentrate (Omega-3; EPAX 1050TG, EPAX AS, containing about 15 % EPA, 40 % DHA, wt/wt; i.e., ~2.8 g EPA + DHA), and (iv) the combination of pioglitazone with EPAX 1050TG (Pio& Omega-3). Randomization was performed using a computer-based algorithm arranging experimental units in blocks of four. The randomization code was kept secret and revealed after the clean-file procedure had been completed when all data had been filled in the case report forms. Placebo and Omega-3 were administered as gelatin-coated 1 g capsules. Thus, the study was double blind for EPA + DHA and open-label for pioglitazone. During week 24, patients were handled similarly as during week 0, except for also performing indirect calorimetry in conjunction with the clamp (see below).

### Anthropometric measurements

Body weight (and height, data not shown) was measured using periodically calibrated scales accurate to 0.1 kg. Waist circumference was measured with a measuring tape placed at the midpoint between the lowest rib and the upper part of the iliac bone (results not shown). Body mass index (BMI) was calculated using the Quetelet formula (weight in kilograms divided by the square of the body height). Blood pressure was measured after 5 min in a seated position at rest, using a digital M6 Comfort monitor (Omron, Kyoto, Japan). Three measurements were taken 2 min apart. The first measurement was discarded, and the mean of the remaining two measurements was recorded.

### Hyperinsulinemic-euglycemic clamp

A 3 h clamp (1 mU/kg.min^−1^), was conducted as described previously [21]. A teflon cannula (Venflon; Viggo, Helsingborg, Sweden) was inserted into an antecubital vein for the infusion of all test substances. A second cannula was inserted into a wrist vein for blood sampling and the hand was placed in a heated (65 °C) box to achieve venous blood arterialization. A stepwise primed-continuous insulin infusion (1 mU/kg body weight.min^−1^ of Actrapid HM; NovoNordisk, Copenhagen, Denmark) was administrated to acutely raise and maintain the plasma concentration of insulin at ~75 μU/ml. Glycemia during the clamps was maintained at approx. 5.5 mmol/l by continuous infusion of 15 % glucose. Arterialized blood glucose concentration was determined every 5–10 min as described in *Other analytical methods* (see below) and the infusion rate was adjusted accordingly. Mean plasma glucose concentrations were comparable within the groups during clamps before and after 24 weeks. The coefficients of variation of glycemia during the studies were less than 5 %. Insulin sensitivity was estimated as the glucose disposal rate (M), i.e. the amount of glucose (mg/kg body weight.min^−1^) needed to maintain the concentration of glucose during the last 20 min of the clamp.

### Indirect calorimetry

At week 24, indirect calorimetry was conducted for 30 min (basal values in fasting state) just before and during the last 30 min of the clamp, using an open-circuit system (VMAX; SensorMedics, Anaheim, CA, USA). Oxygen consumption (*V*O_2_; ml O_2_/min) and carbon dioxide production (*V*CO_2_; ml CO_2_/min) were recorded every 1 min. To assess fuel partitioning, respiratory quotient (RQ; RQ = *V*CO_2_/*V*O_2_) was estimated and substrate utilization and resting energy expenditure (REE) were calculated. Non-oxidative glucose disposal rate (GDR) was calculated by subtracting the rate of glucose oxidation from the total rate of glucose uptake during the last 20 min of the clamp [22].

### Meal test

A meal test was performed as before [21]. After an overnight fast, subjects received a standard breakfast (baguette Crocodille Cheese Gourmet: 180 g, energy 452.8 kcal (1895.7 kJ)) of the following composition: carbohydrates 49 g (45 % energy), proteins 18.5 g (17 % energy), lipids 18.8 g (38 % energy), of which saturated fatty acids 6.8 g, monounsaturated fatty acids 6.0 g, and polyunsaturated fatty acids 5.0 g. Serum concentrations of glucose, immunoreactive insulin, C-peptide, non-esterified fatty acids (NEFA), and triacylglycerol were measured at 0, 30, 60 and 120 min (see below in *Other analytical methods*). Data were expressed as area under the curve (AUC).

### Content of selected fatty acids in serum phospholipids

Serum samples from fasted patients were analyzed using shotgun lipidomics and mass spectrometry, and a sum of concentrations of phospholipids containing EPA and/or DHA divided by total concentration of all phospholipids (Omega-3 PhL Index) was used as a biomarker of EPA and DHA status. Briefly, 50 μl -aliquots of serum were transferred into disposable borosilicate glass tubes with 100 μl of methanol/butylated hydroxytoluene (1,000:1; v/v) containing internal standards for quantification of lipid species: 17:0–17:0 phosphatidic acid, phosphatidylethanolamine, phosphatidylcholine, phosphatidylglycerol, phosphatidylserine, and phosphatidylinositol, respectively (75 nM final concentration). Lipid extracts were prepared using a modified procedure of Bligh and Dyer as previously described [23]. Each lipid extract was diluted with dichloromethane/methanol/isopropanol (1:2:4, v/v/v and 5 mM ammonium acetate) prior to infusion into a mass spectrometer (MS; QTRAP 5500, Sciex, USA; equipped with Turbo V ESI) for the analysis of phospholipids. All the mass spectra and tandem mass spectra were automatically acquired using multiplexed precursor ion (PIS) and neutral loss (NL) scans in positive and negative mode [23]. Analyst 1.6.1/Lipidview 1.3 software was used to identify molecular species and to determine amounts of individual lipids based on internal standard concentrations assuming comparable ionization of standards and phospholipids. Sum formula annotation (e.g. PE 34:2) and acyl chain information coming from negative PIS (e.g. PE 34:2 – PIS 18:2) was used to calculate the additional acyl chain (e.g. PE 34:2 = 18:2 + 16:0). Only combinations of the major fatty acids (carbon:double bonds – 0:0, 12:0, 14:0, 14:1, 16:0, 16:1, 18:0, 18:1, 18:2, 18:3, 20:0, 20:1, 20:2, 20:3, 20:4, 20:5, 22:4, 22:5, 22:6) were used for further data processing. The content of linoleic acid (LA; 18:2 *n*-6) in serum phospholipids was also determined.

### Other analytical methods

Serum glucose levels were analyzed using the Beckman Analyser glucose-oxidase method (Beckman Instruments, Fullerton, CA, USA), plasma immunoreactive insulin and C-peptide concentrations were determined using insulin and C-peptide IRMA kits (Immunotech, Prague, Czech Republic), HbA_1c_, was measured by HPLC (Tosoh, Tokyo, Japan), lipid concentrations were assessed by enzymatic methods (Roche, Basel, Switzerland) and HDL-cholesterol was measured after double precipitation with dextran and MgCl_2_ as described previously [24]. To assess oxidative stress, the amount of lipid peroxidation was determined as thiobarbituric acid reactive substances (TBARS) by the reaction with thiobarbituric acid, the whole blood level of reduced (GSH) and oxidized (GSSG) glutathione was determined with glutathione HPLC diagnostic kit (Chromsystems, Munich, Germany), and the activity of superoxide dismutase (SOD) was analyzed using a superoxide dismutase assay kit (Cayman Chemical, MI, USA). In fasting patients (Table 1), concentrations of serum cytokines were measured using ELISA kits from Biovendor (Czech Republic; total adiponectin, leptin), in the postabsortive state, various cytokines were analyzed by microbead Luminex® assay (Luminex Corporation, Texas, United States; see Additional file 3). Serum pioglitazone levels were estimated using mass spectrometry (see above) [25].

**Table 1:** Basic anthropometric and biochemical characteristics of patients at baseline and after the interventions. The significance threshold for adjusted *p*-values using the Holm-Bonferroni correction is 0.05

	Placebo	Pio	Omega-3	Pio& Omega-3
Age (y)	62.0 (58.0, 65.0)	62.0 (60.0, 65.0)	59.5 (55.8, 63.8)	60.5 (55.3, 65.5)
Systolic blood pressure (mmHg)				
Baseline	138 (129, 149)	142 (136, 151)	146 (135, 153)	139 (131, 145)
Week 24	140 (131, 146)	142 (134, 148)	137 (131, 150)	136 (127, 148)
∆	2 (−3, 7)	1 (−5, 3)	−5 (−8, 2)	−1 (−8, 1)
∆ (%)	1 (−2, 5)	1 (−3, 2)	−3 (−5, 1)	0 (−6, 1)
Diastolic blood pressure (mmHg)				
Baseline	82 (77, 93)	85 (79, 91)	90 (83, 92)	81 (69, 94)
Week 24	85 (70, 90)	82 (73, 89)	86 (75, 90)	82 (76, 92)
∆	1 (−6, 6)	−1 (−9, 2)	0 (−5, 2)	1 (−7, 5)
∆ (%)	1 (−8, 7)	−1 (−8, 2)	0 (−5, 3)	1 (−8, 7)
Body weight (kg)				
Baseline	87.0 (81.2, 103.0)	94.0 (85.0, 102.0)	98.8 (95.2, 110.5)	94.0 (79.5, 107.0)
Week 24	84.0 (79.0, 103.0)	95.0 (88.0, 103.0)	97.5 (91.5, 108.3)	95.5 (83.0, 111.5)
∆	−1.0 (−2.0, 0.0)	1.0 (0.0, 2.8)^c^	−1.5 (−2.6, 1.0)	2.0 (1.0, 3.0)^a, c^
∆ (%)	−1.2 (−2.4, 0.0)	1.3 (0.0, 2.3)^c^	−1.3 (−2.7, 0.8)	1.9 (1.0, 4.2)^a, c^
BMI (kg/m^2^)				
Baseline	30.9 (27.7, 33.5)	32.0 (29.6, 35.4)	34.0 (29.2, 37.5)	31.4 (27.9, 39.8)
Week 24	30.5 (27.4, 32.6)	32.4 (30.2, 36.6)	33.1 (28.7, 37.9)	32.0 (28.7, 40.6)
∆	−0.4 (−0.8, 0.0)	0.4 (0.0, 0.9)^a, c^	−0.5 (−1.0, 0.3)	0.7 (0.4, 1.2)^a, c^
∆ (%)	−1.4 (−2.5, 0.0)	1.3 (0.0, 2.3)^a, c^	−1.3 (−2.7, 0.8)	2.0 (1.1, 4.2)^a, c^
Triacylglycerol (mmol/l)				
Baseline	1.45 (1.23, 1.76)	1.70 (1.17, 2.41)	2.17 (1.50, 2.76)	1.71 (1.32, 2.21)
Week 24	1.41 (1.10, 2.28)	1.89 (1.24, 2.36)	1.67 (1.53, 2.31)	1.52 (1.06, 2.05)
∆	0.21 (−0.13, 0.48)	0.13 (−0.11, 0.66)	−0.48 (−0.73, 0.36)	−0.14 (−0.58, −0.02)
∆ (%)	10.1 (−10.6, 31.3)	4.9 (−13.0, 52.4)	−18.2 (−23.8, 20.2)	−12.2 (−24.0, −1.1)
NEFA (mmol/l)				
Baseline	0.73 (0.64, 0.79)	0.82 (0.64, 1.04)	0.65 (0.54, 0.91)	0.69 (0.53, 0.74)
Week 24	0.65 (0.51, 0.87)	0.51 (0.37, 0.79)	0.60 (0.47, 0.85)	0.53 (0.31, 0.70)
∆	0.21 (−0.13, 0.48)	0.13 (−0.11, 0.66)	−0.48 (−0.73, 0.36)	−0.14 (−0.58, −0.02)
∆ (%)	−11.0 (−40.7, 13.0)	−43.2 (−50.0, −15.4)	−16.0 (−47.6, 25.7)	−9.4 (−43.0, 18.7)
Cholesterol total (mmol/l)				
Baseline	4.60 (4.00, 5.60)	4.54 (3.70, 5.50)	4.70 (4.49, 5.37)	5.25 (4.78, 5.95)
Week 24	4.40 (3.53, 5.32)	4.47 (3.61, 5.06)	4.62 (4.40, 5.12)	4.56 (4.32, 5.45)
∆	−0.47 (−0.62, −0.20)	−0.07 (−0.40, 0.74)	−0.13 (−0.62, 0.21)	−0.23 (−0.87, −0.04)
∆ (%)	−9.8 (−15.9, −4.3)	−2.0 (−7.8, 13.5)	−2.9 (−12.9, 4.5)	−4.7 (−16.4, −0.7)
HDL cholesterol (mmol/l)				
Baseline	1.09 (1.00, 1.29)	1.02 (0.90, 1.16)	1.17 (0.94, 1.28)	1.18 (1.03, 1.28)
Week 24	1.04 (0.79, 1.17)	1.07 (0.96, 1.18)	1.04 (0.91, 1.25)	1.24 (1.10, 1.58)
∆	−0.05 (−0.14, −0.04)	0.07 (−0.05, 0.12)	−0.04 (−0.14, 0.05)	0.12 (0.00, 0.22)
∆ (%)	−4.8 (−10.9, −2.2)	6.2 (−6.5, 8.8)	−2.8 (−14.4, 4.4)	10.5 (0.9, 19.2)
LDL cholesterol (mmol/l)				
Baseline	2.45 (2.00, 3.29)	2.21 (1.70, 2.70)	2.70 (2.28, 3.00)	2.75 (2.37, 3.32)
Week 24	2.60 (1.90, 3.02)	2.60 (1.86, 2.94)	2.81 (2.56, 3.24)	3.03 (2.33, 3.53)
∆	−0.09 (−0.20, 0.00)	0.13 (−0.03, 0.41)	0.16 (−0.17, 0.70)	0.30 (−0.17, 0.65)
∆ (%)	−4.4 (−5.5, 0.0)	7.6 (−1.2, 16.6)	6.8 (−5.6, 27.9)	10.6 (−7.7, 20.2)
SOD (U/ml)				
Baseline	1.40 (0.76, 1.89)	1.44 (0.75, 2.15)	0.90 (0.58, 1.38)	1.16 (0.78, 1.76)
Week 24	1.22 (0.99, 1.76)	1.11 (0.86, 1.99)	0.90 (0.70, 1.35)	0.88 (0.48, 1.25)
∆	0.05 (−0.67, 0.29)	0.21 (−0.33, 0.46)	0.07 (−0.25, 0.46)	−0.23 (−0.68, 0.23)
∆ (%)	2.2 (−33.4, 38.5)	24.3 (−23.0, 43.1)	8.9 (−22.0, 46.1)	−18.0 (−49.5, 34.8)
TBARS (mmol/l)				
Baseline	1.71 (1.33, 2.04)	1.40 (1.16, 2.14)	1.46 (1.35, 2.29)	1.64 (1.26, 2.10)
Week 24	1.42 (1.00, 1.64)	1.36 (0.97, 1.75)	1.12 (0.76, 1.43)	1.28 (0.97, 1.47)
∆	−0.23 (−0.76, 0.28)	−0.10 (−0.42, 0.05)	−0.59 (−0.93, 0.12)	−0.20 (−0.78, 0.09)
∆ (%)	−17.5 (−34.5, 25.8)	−6.7 (−22.6, 3.2)	−43.0 (−56.4, 11.9)	−10.8 (−43.9, 11.2)
Ratio GSSG/GSH				
Baseline	0.17 (0.12, 0.20)	0.14 (0.11, 0.17)	0.11 (0.09, 0.15)	0.11 (0.08, 0.16)
Week 24	0.11 (0.11, 0.15)	0.13 (0.10, 0.14)	0.12 (0.08, 0.18)	0.14 (0.11, 0.20)
∆	−0.04 (−0.09, 0.02)	0.00 (−0.08, 0.03)	0.00 (−0.02, 0.03)	0.01 (−0.02, 0.06)
∆ (%)	−20.9 (−47.5, 19.9)	−3.4(−48.1, 67.3)	2.1 (−19.2, 38.7)	9.0 (−12.7, 53.2)
Leptin (ng/ml)				
Baseline	13.2 (10.6, 17.2)	12.6 (10.0, 26.1)	12.7 (7.0, 23.9)	21.7 (9.7, 30.4)
Week 24	14.4 (7.8, 15.8)	13.0 (9.2, 33.3)	11.3 (7.9, 21.7)	21.0 (8.5, 41.3)
∆	−0.8 (−1.8, 0.6)	2.4 (0.5, 3.0)	−1.1 (−2.9, 1.0)	−0.3 (−1.1, 0.5)
∆ (%)	−3.2 (−14.5, 6.8)	10.3 (1.4, 29.4)	−9.9 (−20.9, 12.2)	−1.9 (−3.8, 3.5)
Adiponectin (μg/ml)				
Baseline	6.3 (5.6, 7.3)	5.7 (4.6, 6.9)	5.3 (4.7, 5.9)	5.6 (4.8, 6.4)
Week 24	6.3 (4.9, 9.0)	9.1 (7.4, 12.8)^a, c^	6.4 (5.7, 7.2)	8.9 (7.5, 9.9)^a, c^
∆	0.4 (0.0, 1.0)	3.5 (2.6, 5.0)^a, c^	0.9 (0.6, 1.2)	3.7 (2.6, 4.8)^a, c^
∆ (%)	9.4 (−0.2, 27.4)	60.0 (45.4, 73.4)^a, c^	15.8 (9.0, 26.9)	64.7 (44.6, 91.8)^a, c^
Omega-3 PhL Index				
Baseline	4.9 (4.3, 5.8)	5.2 (4.4, 5.8)	4.9 (4.6, 5.5)	5.3 (4.1, 5.7)
Week 24	4.4 (4.1, 4.8)	4.5 (4.2, 5.2)	8.2 (7.1, 8.9)^a, b^	8.3 (7.4, 9.2)^a, b^
∆	−0.1 (−0.5, 0.2)	−0.3 (−0.7, 0.2)	2.8 (2.1, 4.4)^a, b^	3.4 (2.5, 3.6)^a, b^
∆ (%)	−2.6 (−10.5, 4.6)	−5.2 (−13.9, 2.7)	60.8 (43.9, 88.5)^a, b^	62.8 (41.4, 99.9)^a, b^

Data represent a median and interquartile range (Q1, Q3). Various parameters were analyzed in overnight (8–10-h) fasting patients at baseline and at week 24 of the respective interventions. ∆, a difference between week 24 and baseline values; ∆ (%), a difference between week 24 and baseline values in % of the baseline value; BW, body weight; BMI, body mass index, NEFA, non-esterified fatty acids; SOD, superoxide dismutase; TBARS, thiobarbituric acid reactive substances; GSSH, oxidized glutathione; GSH, reduced glutathione. ^a, b, c^Significant differences compared with Placebo, Pio, and Omega-3, respectively

### Study endpoints

The primary endpoints were changes from baseline in (i) insulin sensitivity (M; hyperinsulinemic-euglycemic clamp) and (ii) fasting triacylglycerol levels at week 24. Secondary endpoints included the changes in fasting glycemia and HbA_1c_, and postprandial change in glucose, NEFA and triacylglycerol levels (meal test), metabolic flexibility assessed by indirect calorimetry, and inflammatory markers.

### Statistical analysis

A power calculation indicated that 16 patients were needed to detect a 7 % difference in M due to the intervention with the probability 1 at the 0.05 level of significance (assuming accuracy of measurement 5 %). All values are presented as median and interquartile range (IQR). Data from the baseline and the end of the study, and the changes (∆) between the baseline and the end at week 24, were analyzed by the Kruskal-Wallis one-way analysis of variance (ANOVA) using SigmaStat 3.5 (SSI, San Jose, CA, USA) and the statistical software R version 3.1.0 (http://www.r-project.org). The Holm–Bonferroni corrections for multiple comparisons were used. Wilcoxon signed-rank test was used to analyze the effect of intervention within each subgroup. Threshold of significance was defined at a *p* value of ≤ 0.05. For the analysis of the dependence of the response (∆ value) of selected variables on the corresponding change in Omega-3 PhL Index (∆ Omega-3 PhL Index), a linear regression model with dummy variables that indicate a subgroup Placebo, Pio, Omega-3, and Pio& Omega-3, respectively, was used. Models were considered with interactions since the effect of ∆ Omega-3 PhL Index varies by subgroups, so ∆ Omega-3 PhL Index and subgroups interact in affecting ∆ value of selected variables.

## Results

### Basal characteristics

Of the 69 patients enrolled, data from 60 patients could be used for the final evaluation (Fig. 1). Thus, 5 patients withdrew owing to personal reasons; and after the intervention started, 1 patient was excluded due to failure to adhere to the study protocol. Based on serum pioglitazone measurements (not shown), 3 patients were excluded due to detection of pioglitazone already at the baseline, while the pioglitazone levels assessed at week 24 confirmed adherence to the study protocol in the Pio and Pio& Omega-3 subgroups.

No significant differences were observed between the subgroups in basic anthropometric and biochemical characteristics measured in the fasting state during the study (Table 1). In the subgroup analysis by the effect of the 24-week-intervention (see ∆-values in Table 1), both Pio and Pio& Omega-3 increased body weight and BMI compared with Placebo or Omega-3 (except for Placebo vs. Pio in the case of body weight; *p* = 0.10). Triacylglycerol, NEFA and total cholesterol in serum, blood lipoproteins including HDL cholesterol and LDL cholesterol, and markers of oxidative stress in serum including SOD activity, TBARS and GSSG/GSH ratio were not significantly affected by any of the interventions (Table 1). Magnetic resonance spectroscopy did not show any significant effect of the interventions on the ectopic lipid content (see Additional file 1). Leptin levels were increased in Pio compared with the other subgroups (*p* = 0.02). At week 24, adiponectin levels were higher in Pio and Pio& Omega-3 than in Placebo subgroup, reflecting ~1.6-fold fold stimulatory effect in the median concentration in both Pio and Pio& Omega-3 (*p <* 0.0001). Adiponectin levels in the Omega-3 subgroup were higher, reflecting 1.2-fold increase in the median in this subgroup (*p <* 0.001) but were unchanged in the Placebo subgroup (Table 1).

**Fig. 2:**
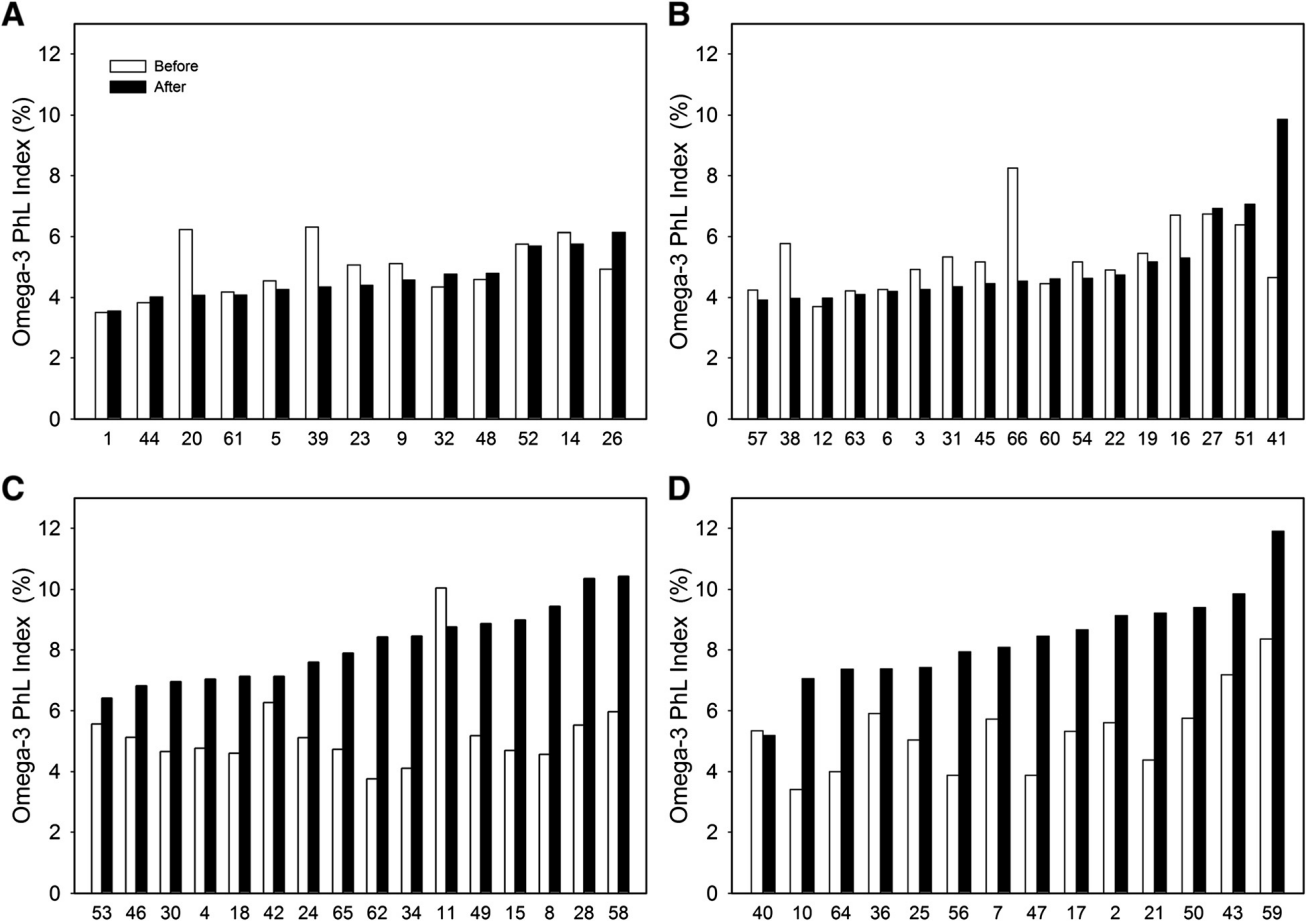
Omega-3 PhL Index in individual patients. Analysis was performed at baseline (*white bars*) and at week 24 (*black bars*) in Placebo (**a**) Pio (**b**) Omega-3 (**c**) and Pio& Omega-3 (**d**) subgroups. Case numbers are indicated

Omega-3 PhL Index was similar in all subgroups at the baseline, while at week 24 it was not affected by either Placebo or Pio, but increased to a similar extent (1.6–1.7-fold; *p* < 0.0001 and *p* = 0.0001, respectively) in response to both Omega-3 and Pio& Omega-3 (Table 1). As observed before [26], Omega-3 PhL Index differed between individuals, showing up to ~3-fold differences when all 60 patients were compared at the baseline (Fig. 2 a-d). In the EPA + DHA supplemented subgroups (Omega-3 or Pio& Omega-3) a maximum ~2.2-fold difference in the median Omega-3 PhL Index was observed between individuals at the end of the intervention (Table 1). The variable increase in the Omega-3 PhL Index in response to EPA + DHA supplementation was independent of the pre-intervention value (Fig. 2 c, d). In contrast with the Omega-3 PhL Index (i.e., the EPA and DHA in serum phospolipids), LA content in phospholipids was not affected by any intervention and no differences in LA content between the subgroups of patients were found, either before or after the intervention (see Additional file 2).

### Glucose metabolism

No differences between Placebo, Pio and Pio& Omega-3 subgroups were observed in the markers of acute and long-term glycemic control, i.e., fasting blood glucose and serum HbA_1c_ level, respectively, either at baseline, or at week 24 (Table 2). In response to Omega-3, both parameters increased by ~1.2-fold (fasting blood glucose, *p* = 0.02; HbA_1c_, *p* = 0.01) resulting in a significant effect of Omega-3 (see ∆-values in Table 2) and indicating a marginal deterioration of glycemic control. Glucose disposal rate, assessed using hyperinsulinemic-euglycemic clamp (M value), is a measure of insulin sensitivity. While it was similar in all the subgroups at baseline, M increased (*p* = 0.04) in Pio& Omega-3 subgroup during the intervention and at week 24, it was higher in the Pio& Omega-3 compared with the Omega-3 subgroup. In the subgroup analysis by the effect of the intervention (see ∆-values in Table 2), the effect of Pio (*p* = 0.12) and Pio& Omega-3 (*p* = 0.12) tended to be different from that of Omega-3. Thus, regarding the effects on insulin sensitivity, Omega-3 exerted a neutral effect compared with Placebo, while the results collectively document a marginal improvement by the Pio& Omega-3 intervention.

**Table 2:** Glucose homeostasis and insulin sensitivity at baseline and after the intervention. The significance threshold for adjusted *p*-values using the Holm-Bonferroni correction is 0.05

	Placebo	Pio	Omega-3	Pio& Omega-3
HbA_1c_ (IFCC, mmol/mol)				
Baseline	52 (48, 56)	52 (47, 54)	50 (47, 55)	49 (44, 53)
Week 24	49 (48, 51)	49 (46, 55)	58 (51, 73)	48 (46, 53)
∆	0 (−5, 1)	0 (−5, 1)	7 (1, 13)^a, b^	0 (−3, 2)
∆ (%)	0.0 (−8.9, 2.1)	0.0 (−7.6, 1.9)	14.7 (2.6, 22.1)^a, b^	0.0 (−6.2, 3.9)
Fasting blood glucose (mmol/l)				
Baseline	7.31 (6.40, 8.27)	7.48 (7.15, 8.47)	7.67 (6.53, 8.87)	7.10 (6.51, 8.18)
Week 24	7.26 (5.99, 8.17)	7.39 (6.79, 8.00)	9.10 (7.34, 10.50)	7.22 (6.59, 8.44)
∆	−0.10 (−1.32, 0.80)	−0.25 (−0.78, 0.01)	1.07 (0.18, 2.02)^b^	0.05 (−0.51, 0.47)
∆ (%)	−1.2 (−18.1, 12.4)	−3.7 (−8.4, 0.1)	17.0 (1.6, 25.2)^b^	0.8 (−5.6, 7.4)
M (mg/kg.min^−1^)				
Baseline	3.42 (2.38, 3.97)	2.51 (2.12, 4.10)	2.66 (2.15, 3.34)	3.21 (2.52, 3.68)
Week 24	2.79 (1.89, 3.09)	2.93 (2.69, 4.47)	2.33 (1.18, 3.41)	3.55 (3.08, 4.34)^c^
∆	−0.60 (−1.24, 0.60)	0.29 (−0.34, 1.30)	−0.58 (−1.25, −0.13)	0.53 (−0.20, 0.92)
∆ (%)	−17.1 (−38.1, 17.2)	12.0 (−13.0, 52.7)	−21.2 (−41.0, 1.9)	14.6 (−6.2, 40.3)

Data represent a median and interquartile range (Q1, Q3). Glycemia and plasma HbA_1c_ levels were measured in overnight (8–10-h) fasting patients, while glucose disposal rate (M) was evaluated using hyperinsulinemic-euglycemic clamp at baseline and at week 24. ∆, a difference between week 24 and baseline values, ∆ (%), a difference between week 24 and baseline values in % of the baseline value. ^a, b, c^Significant differences compared with Placebo, Pio, and Omega-3, respectively

### Energy metabolism and metabolic flexibility

At week 24, indirect calorimetry was performed in conjunction with the hyperinsulinemic-euglycemic clamp. None of the measured parameters, namely REE, RQ, carbohydrate oxidation and fat oxidation were significantly different between the subgroups before or during the clamp. Non-oxidative GDR, assessed during the clamp, was also similar in all subgroups (Table 3).

**Table 3:** Energy metabolism and substrate utilization after the intervention. The significance threshold for adjusted *p*-values using the Holm-Bonferroni correction is 0.05

	Placebo	Pio	Omega-3	Pio& Omega-3
REE (kcal/day)	1585 (1410, 2157)	1611 (1534, 1816)	1780 (1653, 2000)	1566 (1503, 1733)
RQ				
Fasting state	0.80 (0.77, 0.83)	0.79 (0.77, 0.85)	0.77 (0.74, 0.83)	0.80 (0.76, 0.83)
During clamp	0.85 (0.81, 0.91)	0.87 (0.83, 0.91)	0.86 (0.83, 0.87)	0.89 (0.84, 0.91)
Diff C-F	0.07 (0.02, 0.08)	0.06 (0.03, 0.09)	0.08 (0.02, 0.11)	0.07 (0.06, 0.10)
Carbohydrate oxidation (mg/kg.min^−1^)				
Fasting state	0.77 (0.42, 1.18)	0.78 (0.47, 1.39)	0.44 (0.17, 0.92)	0.66 (0.43, 1.27)
During clamp	1.09 (0.65, 1.99)	1.15 (0.88, 1.51)	1.17 (1.02, 1.32)	1.50 (1.17, 1.73)
Diff C-F	0.59 (0.28, 0.79)	0.51 (0.00, 0.71)	0.90 (0.29, 1.13)	0.77 (0.41, 0.82)
Fat oxidation (mg/kg.min^−1^)				
Fasting state	0.80 (0.52, 0.88)	0.70 (0.31, 0.80)	0.91 (0.50, 1.01)	0.61 (0.48, 0.89)
During clamp	0.43 (0.29, 0.73)	0.34 (0.24, 0.52)	0.46 (0.34, 0.61)	0.32 (0.13, 0.47)
Diff C-F	−0.38 (−0.43, −0.1)	−0.20 (−0.39, −0.08)	−0.31 (−0.54, −0.10)	−0.35 (−0.40, −0.24)
Non-oxidative GDR (mg/kg.min-1)				
	1.47 (0.87, 2.00)	1.80 (1.28, 2.51)	0.60 (0.05, 1.74)	2.32 (1.46, 2.81)

**Fig. 3:**
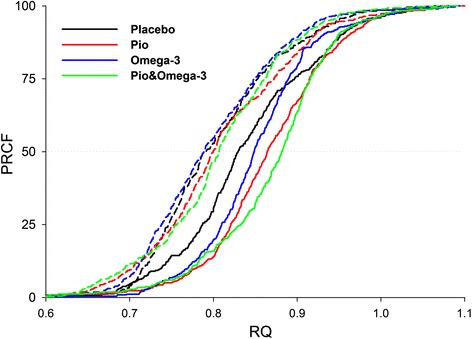
Metabolic flexibility after the interventions. RQ data from indirect calorimetry at week 24 (see Table 3) were used to construct PRCF curves, each of which represents data (~400) pooled from all patients in the given subgroup either in fasting state (*dashed lines*) or during clamp (*solid lines*). RQ values corresponding to EC_50_ (50^th^ percentile value) on each of the curves were obtained and the difference between this RQ value assessed during the clamp and fasting, respectively, was used as a marker of metabolic flexibility to glucose (PRCF curve shift; Placebo, 0.04; Pio, 0.06; Omega-3, 0.06; Pio& Omega-3, 0.07)

Data represent a median and interquartile range (Q1, Q3). Data were obtained using indirect calorimetry performed in conjunction with hyperinsulinemic-euglycemic clamp at week 24 (see Table 2). Differences between parameters measured during the clamp and fasting state are also shown (Diff C-F). REE, resting energy expenditure; RQ, respiratory quotient; GDR, glucose disposal rate (indirect calorimetry). For evaluation of the effect of respective interventions on RQ (metabolic flexibility), see also Fig. 3. Non-oxidative glucose GDR was calculated as described in Methods. No significant differences between subgroups were found

Next, we attempted to detect possible differences in metabolic flexibility between the subgroups at week 24. We focused on the increase in RQ during the clamp, as a common way for assessment of metabolic flexibility to carbohydrates, which is usually impaired in insulin-resistant individuals [27]. No significant differences in the increase in median RQ between the subgroups were observed (Table 3). Therefore, a robust approach based on the evaluation of percent relative cumulative frequency (PRCF) curves of RQ values was used while pooling all RQ values from all the patients within subgroups. This was done for both fasting and clamp periods (Fig. 3). Provided that the PRCF curve represents normally distributed data, the value of EC_50_ of PRCF (50^th^ percentile) corresponds to a mean RQ value, while these curves also allow to identify differences that may exist at either lower or upper levels of RQ range [28]. During the clamp, PRCF curves within each subgroup shifted to the right (i.e., towards glucose oxidation), documenting various degrees of metabolic flexibility to glucose. Compared to Placebo, metabolic flexibility was improved by all interventions in the following order of effect: Pio < Omega-3 < Pio& Omega-3 (see the PRCF curve shifts in the legend to Fig. 3).

### Postprandial metabolism

**Fig. 4:**
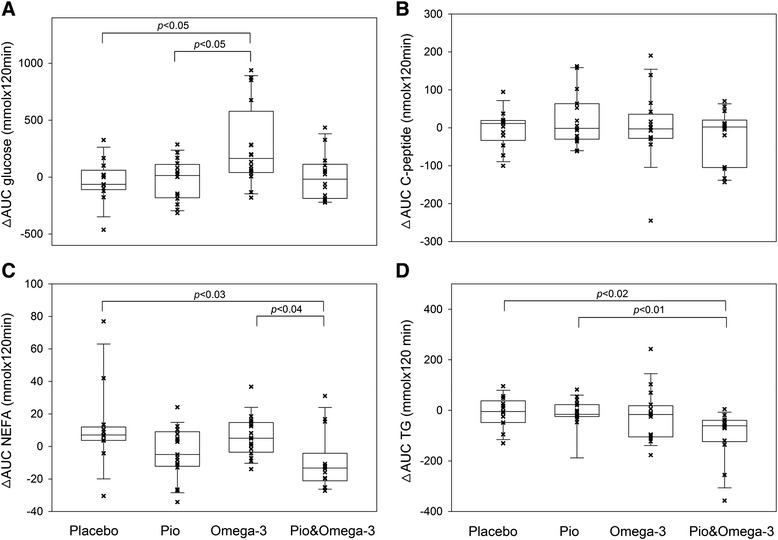
The effects of interventions on postprandial metabolism of selected metabolites and insulin response. A meal test was performed in overnight fasted patients at baseline and at week 24. Transient postprandial increases in serum concentrations of various analytes were evaluated during 120 min following a standard breakfast. The difference between total AUC for each analyte measured at week 24 and baseline (∆AUC) is shown for glucose (**a**) C-peptide (**b**) NEFA (**c**) and triacylglycerol TG; (**d**). Plots represent 10^th^, 25^th^, 50^th^ (median), 75^th^, and 90^th^ percentiles. Significant differences (Kruskal-Wallis test) between the subgroups are indicated

A meal test was performed at baseline and at week 24, allowing for assessment of the effects on postprandial metabolism of both glucose and lipids, and of the insulin response to a carbohydrate load. Transient increases in serum glucose, C-peptide, NEFA and triacylglycerol (and insulin; not shown) levels triggered by a standard breakfast were followed during 120 min. Data were expressed as AUC (Fig. 4). At baseline, no significant differences between the subgroups were observed; at week 24, Pio& Omega-3 subgroup showed faster metabolism of triacylglycerol (lower AUC) compared with both Placebo and Omega-3 (*p* = 0.04; not shown). In the subgroup analysis by the effect of the intervention, several significant results were obtained. Thus, the difference between AUC at week 24 and baseline (∆AUC) for glucose was higher in Omega-3 compared with the other subgroups (Fig. 4a; except for Omega-3 vs. Pio& Omega-3*, p* = 0.06), suggesting a deterioration of glucose metabolism. In the case of C-peptide (and insulin; not shown), no significant differences between the interventions were found (Fig. 4b). Postprandial metabolism of NEFA was accelerated in response to Pio& Omega-3, as documented by the lower ∆AUC in this subgroup compared with both Placebo and Omega-3 (Fig. 4c). Regarding the metabolism of triacylglycerols (Fig. 4d), ∆AUC was similar in the Placebo, Pio and Omega-3 subgroup, while it was decreased in response to Pio& Omega-3.

Previous studies showed an anti-inflammatory effect of EPA + DHA at the level of some plasma cytokines in the postprandial state [29]. Therefore, various anti-inflammatory (IL-1RA and IL-10) and pro-inflammatory cytokines (MCP-1, CRP, TNF-α and IL-6), as well as proteins involved in cell adhesion (sVCAM-1, sICAM-1, sE-selectin, sP-selectin, sPECAM-1) and neovascularization (sCD105) were evaluated in serum samples collected at 120 min of the test, both at baseline and week 24. Except for sP-selectin, no significant effects of the interventions were observed (see Additional file 3).

## Discussion

We show here that a 6-month-combined intervention using a relatively low dose of pioglitazone and a dose of EPA + DHA, which is within the range that is approved for treatment of hypertriacylglycerolemia [13], exerts additive beneficial effects on metabolism of both NEFA and triacylglycerols in T2D patients (Fig. 4). All the patients were already receiving metformin therapy and well compensated regarding glycemic control at the baseline, and most of them were obese, thus representing a typical population of patients treated for T2D.

In spite of the triacylglycerol-lowering effect of EPA + DHA, shown in many studies, including those in patients with T2D, as well as the hypolipidemic effects of thiazolidinediones (see Background), fasting serum triacylglycerol levels were not significantly affected by any of the studied interventions. It is likely that the background metformin therapy, which was shown to improve dyslipidemia in patients with T2D [30], could mask the effect of the tested interventions. This is also consistent with the notion that most of the studies demonstrating the triacylglycerol-lowering effect of EPA + DHA in the patients with T2D were performed before the beginning of the metformin era as well as the use of thiazolidinedione therapy of these patients. Moreover, metformin could also mask the additive triacylglycerol-lowering effects of EPA + DHA in T2D dyslipidemic patients under statin therapy, which was observed before [31] but not in our study (not shown). Nevertheless, the additive improvement in metabolism of both NEFA and triacylglycerols by the combined intervention found using the meal test in this study (Fig. 4) suggests that increased intake of EPA + DHA could reduce the cardiovascular risk even in T2D patients treated with metformin. This complex effect of the combined intervention is of clinical relevance because increased postprandial triacylglycerolemia represents an independent risk factor of cardiovascular disease in T2D patients [10, 17]. The mechanisms behind the effect of the combined intervention on metabolism of NEFA and triacylglycerols require clarification. It is likely that PPARα-mediated catabolism of fatty acids [12] and/or their trapping in adipose tissue, i.e., the biochemical activities that are possibly altered in the patients (reviewed in [32]), could contribute to the NEFA-lowering effect. Regarding the beneficial effect on metabolism of triacyglycerols, depression of the rate of VLDL-triacyglycerol secretion from the liver should be considered [7, 33].

That all the patients were well-controlled under metformin therapy could also affect other results of the study. First, it could explain why no effect on proteins linked to inflammation (anti – inflammatory cytokines, namely IL-1RA, IL-10, and pro-inflammatory cytokines, namely MCP-1, CRP, TNF-α, IL-6) was observed (Additional file 3), since metformin is known to exert anti-inflammatory action [3]. Indeed cytokine and adhesion molecule concentrations (cVCAM-1, sICAM-1, sE-selectin, sPECAM-1) were similar to those reported for healthy male subjects of different ages [34]. Importantly, no deleterious effects of any of the interventions on the markers of oxidative stress (SOD, TBARS, ratio GSSG/GSH) were observed (Table 1), possibly, due to the antioxidant effect of metformin [4]. Similarly, also insulin secretion remained unaffected by the interventions (see Results, *Postprandial metabolism*).

**Fig. 5:**
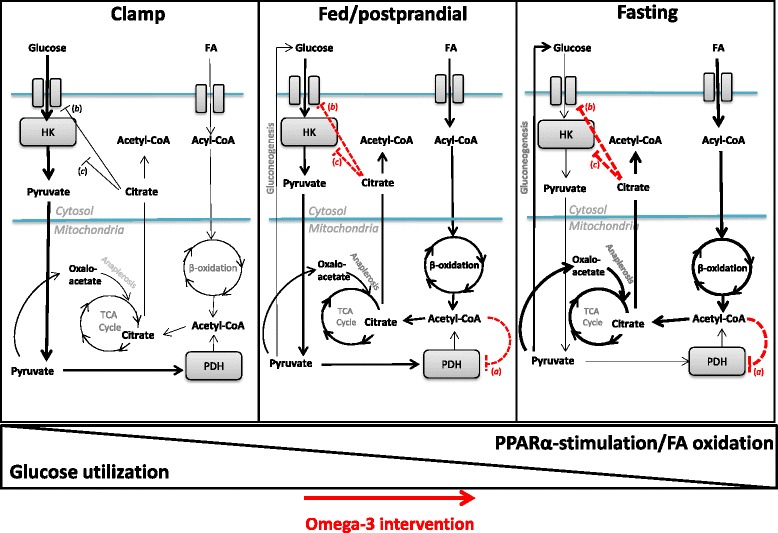
The effects of omega-3 fatty acids on glucose metabolism in insulin-sensitive tissues depend on energy fuels. When glucose serves as the major energy substrate (Clamp), glucose utilization is only marginally affected. With increased postprandial intake of both carbohydrates and lipids (Fed/postprandial), or when fatty acids (FA) serve as the major fuel (Fasting), β-oxidation is stimulated by EPA + DHA *via* PPARα-signaling [12], which results in reduced glucose utilization (red dashed lines) by several mechanisms involving the Randle cycle (55). Inhibition of glucose oxidation at the level of pyruvate dehydrogenase (PDH) by acetyl-CoA (*a*) leads also to rerouting of pyruvate to anaplerosis (muscle) and/or gluconeogenesis (liver); citrate accumulation in the cytosol results in inhibition of glucose uptake (*b*) and inhibition of glycolysis (*c*) at the level of hexokinase (HK)

Second, only limited additional benefits of pioglitazone and/or Omega-3 may be expected in metformin treated T2D patients. In fact, Omega-3 alone marginally impaired markers of glycemic control (HbA_1c_ levels and fasting glycemia; Table 2) and glucose metabolism in the meal test (Fig. 4), but did not diminish insulin sensitivity (M) evaluated using a hyperinsulinemic-euglycemic clamp (Table 2). These results are compatible with a model in which EPA + DHA *per se* do not deteriorate glucose utilization when glucose represents the main energy fuel (Fig. 5, Clamp). However, when the supply of both carbohydrates and lipids is increased (e.g., during the meal test; Fig. 5, Fed), or when fatty acids liberated from adipose tissue represent the main energy fuel (Fig. 5, Fasting), glucose utilization is inhibited by multiple mechanisms involved in the Randle cycle (reviewed in [35]) reflecting the PPARα-mediated stimulation of fatty acid oxidation by EPA + DHA [12]. This would lead to the observed subtle deterioration of glucose metabolism by Omega-3. Metabolic changes in skeletal muscle, the main site of glucose utilization, probably play a major role. That fasted glycemia is selectively increased by Omega-3 (Table 2) could also reflect increased hepatic gluconeogenesis stimulated in face of enhanced fatty acid oxidation [36]. Both decreased postprandial metabolism of glucose (Fig. 4) and elevated glycemia in fasted state could contribute to raised HbA_1c_ levels in the Omega-3 subgroup (Table 2). Thus, our results also help to clarify some controversies regarding the effects of EPA + DHA on glucose homeostasis in T2D patients (see Background).

The negative effects of Omega-3 on glycemic control and glucose metabolism were prevented by Pio. Insulin sensitivity was increased by Pio&Omega-3, and tended to be improved by Pio compared with Omega-3 (Table 2), which is also in agreement with the induction of adiponectin by both Pio& Omega-3 and Pio (Table 1). These results were consistent with changes in metabolic flexibility to glucose evaluated using indirect calorimetry during the hyperinsulinemic-euglycemic clamp at week 24 (Fig. 3), since this parameter closely reflects whole-body glucose uptake [27]. Thus, all the interventions, and especially Pio& Omega-3, increased metabolic flexibility. These results are consistent with the above model and also with our previous study showing additive improvement in metabolic flexibility [20] and insulin sensitivity [7] by combined interventions using EPA + DHA and thiazolidinediones in dietary obese mice. In both the animal experiments and the present clinical trial, the robust PRCF analysis of RQ was used. This approach, which revealed here subtle differences in metabolic flexibility, has not been applied in humans before.

Few studies were conducted to characterize possible modulation of metabolic flexibility by EPA + DHA in T2D patients, and very little is known about the effects of combined interventions using EPA + DHA and pharmaceuticals. It has been shown that EPA + DHA administered as a 4-h lipid infusion resulted in a marginal improvement of metabolic flexibility without affecting insulin sensitivity [37]. Over a 9-week-period, dietary EPA + DHA exerted a transient improvement of glucose utilization followed by a shift from glucose to lipid catabolism, but the effect on metabolic flexibility is difficult to assess from these data since a relatively large volume (20 ml) of crude fish oil containing different lipid fractions besides EPA + DHA was used [38]. Thus, our study is unique regarding the use of a complex methodological approach including the indirect calorimetry, clamp as well as a meal test, which allowed us to demonstrate the additive improvements in metabolic flexibility to glucose, and namely in the postprandial lipid metabolism, by pioglitazone in combination with highly purified EPA + DHA.

Evaluation of serum levels of both pioglitazone and EPA + DHA increased the power of the study by controlling the adherence to the therapy, and enabled a more detailed analysis of the measured parameters relative to the changes in Omega-3 PhL Index. However, only weak correlations (*p* < 0.1) were detected when an increase in HbA_1c_ levels or a decrease in NEFA levels was examined (see Additional file 4). Further studies are needed to analyze the mechanisms behind the metabolic effects of interventions observed in our study, including the evaluation of changes in muscle glycogen content during the clamp. In this context, muscle glycogen was measured only in the fasting state and no differences between the subgroups were found (Additional file 1).

In spite of the fact that pioglitazone is a well-established pharmaceutical with lasting insulin-sensitizing effects, and despite its other beneficial effects in patients with T2D, its clinical use has declined recently due to the risk of the side-effects (see Background). Importantly, at least some of these concerns have been disproved recently. Namely, it has been demonstrated that the cumulative use of pioglitazone or rosiglitazone was not associated with the incidence of bladder cancer [39]. The results of our study document beneficial effects of a relatively low dose of pioglitazone on lipid metabolism when pioglitazone was used as part of the combined intervention with *n*-3 fatty acids. This observation is relevant for reducing the risk of the side-effects of pioglitazone under clinical settings.

## Conclusions

In overweight/obese T2D patients on stable metformin therapy, and in spite of the modest negative effect of Omega-3 on glycemic control and postprandial glucose metabolism, no adverse effect on insulin sensitivity was observed. In response to the combined intervention using Pio& Omega-3, the negative effect of Omega-3 on glucose metabolism was avoided, insulin sensitivity increased, and lipid metabolism was additively improved. Thus, typical T2D patients may be advised to increase their EPA + DHA intake, either in the form of dietary supplements or sea food and fish, in order to increase the efficacy of pharmacotherapies and to prevent diseases linked to inflammation as well as cardiovascular disease, providing that glycemic control is closely monitored. EPA + DHA are likely to bring benefits on cardiovascular health of diabetic patients.

## Additional files

Additional file 1:
**Liver and muscle lipid content before and after the intervention and muscle glycogen content after the intervention.** (DOCX 44 kb) Additional file 2:
**The content of linoleic acid (LA) in serum phospholipids.** (DOCX 94 kb) Additional file 3:
**Serum levels of inflammatory markers in the postprandial state.** (DOCX 39 kb) Additional file 4:
**Correlations between changes in Omega-3 PhL Index and changes in selected variables in response to interventions.** (DOCX 22909 kb)

## Abbreviations

∆: difference between week 24 and baseline values
AUC: area under the curve
BMI: body mass index
DHA: docosahexaenoic acid
EPA: eicosapentaenoic acid
GDR: glucose disposal rate (indirect calorimetry)
GSH: reduced glutathione
GSSG: oxidized glutathione
HbA_1c_: hemoglobin A_1c_
IL: interleukin
IQR: interquartile range
LA: linoleic acid (18:2 *n*-6)
M: glucose disposal rate (hyperinsulinemic-euglycemic clamp)
NEFA: non-esterified fatty acids
Omega-3: EPA + DHA concentrate (5 g/day) supplementation
Omega-3 PhL Index: relative concentration of EPA + DHA in plasma phospholipids
Pio: pioglitazone (15 mg/day) intervention
Pio& Omega-3: pioglitazone (15 mg/day) intervention combined with EPA + DHA concentrate (5 g/day) supplementation
PPAR: peroxisome proliferator-activated receptor
PRCF: percent relative cumulative frequency
REE: resting energy expenditure
RQ: respiratory quotient
SOD: superoxide dismutase
T2D: type 2 diabetes
TBARS: thiobarbituric acid reactive substances
*V*CO_2_: carbon dioxide production
*V*O_2_: oxygen consumption
