# Supplementary material for: Combined intervention with pioglitazone and n-3 fatty acids in metformin-treated type 2 diabetic patients: improvement of lipid metabolism
Source: Nutr Metab (Lond). 2015 Dec 2;12:52. doi: 10.1186/s12986-015-0047-9 (PMC4667423; doi:10.1186/s12986-015-0047-9)
Supplement: Additional file 1: — Liver and muscle lipid content before and after the intervention and muscle glycogen content after the intervention. (DOCX 44 kb) [file 12986_2015_47_MOESM1_ESM.docx]

**Additional file 1. Liver and muscle lipid content before and after the intervention and muscle glycogen content after the intervention.**

The significance threshold for adjusted *p*-values using the Holm-Bonferroni correction is 0.05.

|  | Placebo | Pio | Omega-3 | Pio&Omega-3 |
| --- | --- | --- | --- | --- |
| Liver lipid content (%) |  |  |  |  |
| Baseline | 8.29 (5.00, 14.89) | 10.90 (8.14, 15.25) | 11.29 (6.09, 17.40) | 11.03 (9.83, 16.31) |
| Week 24 | 8.79 (5.04, 10.88) | 9.84 (5.86, 11.84) | 12.00 (5.92, 19.37) | 12.07 (9.93, 14.95) |
| ∆ | -0.47 (-2.97, 0.94) | -2.78 (-4.38, 1.41) | -0.62 (-4.28, 0.67) | -1.10 (-4.67, 1.14) |
| ∆ (%) | -0.4 (-18.4, 32.4) | -25.1 (-31.8, 10.9) | -17.4 (-28.0, 19.3) | -13.9 (-28.5, 9.0) |
| Muscle lipids |  |  |  |  |
| CH_2_/Cr signal |  |  |  |  |
| Baseline | 4.67 (3.36, 5.54) | 4.04 (3.12, 6.62) | 4.04 (3.13, 6.31) | 3.58 (2.70, 4.29) |
| Week 24 | 4.65 (3.58, 6.11) | 4.23 (3.06, 5.42) | 5.27 (3.21, 6.37) | 2.89 (1.97, 4.67) |
| ∆ | 0.61 (-0.46, 0.77) | 0.16 (-3.20, 1.20) | 0.01 (-0.32, 0.53) | -0.16 (-1.31, 1.25) |
| ∆ (%) | 12.5 (-12.1, 27.6) | 8.5 (--42.3, 28.1) | 0.1 (-8.1, 10.9) | -8.1 (-37.8, 46.0) |
| CH_3_/Cr signal |  |  |  |  |
| Baseline | 0.96 (0.59, 1.25) | 0.74 (0.56, 1.27) | 0.87 (0.52, 1.35) | 0.55 (0.49, 0.75) |
| Week 24 | 0.86 (0.59, 1.17) | 0.74 (0.60, 1.00) | 0.95 (0.69, 1.23) | 0.48 (0.32, 0.78) |
| ∆ | -0.05 (-0.20, 0.10) | 0.09 (-0.56, 0.14) | -0.03 (-0.13, 0.11) | -0.10 (-0.16, 0.18) |
| ∆ (%) | -4.0 (-18.3, 10.4) | 6.8 (-45.0, 26.9) | -2.2 (-16.2, 22.5) | -18.4 (-26.1, 19.7) |
| Double bond per fatty acid chain | | | | |
| Baseline | 0.76 (0.63, 1.43) | 1.45 (0.72, 2.80) | 0.98 (0.80, 1.23) | 1.23 (0.69, 3.09) |
| Week 24 | 0.68 (0.54, 1.06) | 0.66 (0.47, 1.27) | 0.96 (0.53, 1.66) | 1.04 (0.69, 1.93) |
| ∆ | -0.26 (-0.47, 0.25) | -0.34 (-2.05, 0.54) | 0.04 (-0.74, 0.57) | -0.41 (-2.40, 0.37) |
| ∆ (%) | -21.9 (-47.9, 31.1) | -40.4 (-74.4, 53.9) | 7.6 (-63.1, 56.4) | -31.1 (-44.1, 56.3) |
| Muscle glycogen | |  |  |  |
|  | 312 (271, 391) | 317 (311, 360) | 328 (299, 362) | 281 (267, 316) |

Data are a median and interquartile range (Q1, Q3). Lipid content in liver and intramyocellular lipid content in skeletal muscle (*m. tibialis*) was evaluated using magnetic resonance spectroscopy as previously described [[1](#_ENREF_1), [2](#_ENREF_2)]. ∆, a difference between week 24 and baseline values. ∆ (%), a difference between week 24 and baseline values in % of the baseline value. CH_3_- signal of terminal ‑CH_3_ group of FA chain, CH_2_ – signal of ‑CH_2_‑ groups (except of CH_2_ groups adjacent to ‑CH=CH‑ or to –CO- group). Cr - internal signal standard (total signal of creatine and phosphocreatine). For the estimation of muscle glycogen, samples of *m.* *vastus lateralis* weighting 50–100 mg, were obtained by muscle biopsy from overnight-fasted subjects, using the Bergstrom technique [[3](#_ENREF_3)] and snap frozen and stored in liquid nitrogen. Frozen muscle samples were freeze-dried and dissected from visible blood, connective tissue and fat using a binocular microscope [[4](#_ENREF_4)]. The cleaned samples were homogenized in ice-cold buffer containing 50 mM HEPES, 150 mM NaCl, 10 mM Na_4_P_2_O_7_, 30 mM NaF, 1 mM Na_3_VO_4_, 10 mM EDTA, 2.5 mM benzamidine, pH 7.4 (1:125 dry weight/volume). The homogenates were hydrolyzed in 1.8 M HCl for 2.5 h at 100°C as described [[5](#_ENREF_5)]. Glycogen content was determined fluorometrically [[6](#_ENREF_6)] and expressed as mmol glucose units/kg dry weight. No significant differences between subgroups were found.

**References**

1. Skoch A, Jiru F, Dezortova M, Krusinova E, Kratochvilova S, Pelikanova T, Grodd W, Hajek M: Intramyocellular lipid quantification from 1H long echo time spectra at 1.5 and 3 T by means of the LCModel technique. J Magn Reson Imaging 2006; 23:728-735.

2. Hajek M, Dezortova M, Wagnerova D, Skoch A, Voska L, Hejlova I, Trunecka P: MR spectroscopy as a tool for in vivo determination of steatosis in liver transplant recipients. MAGMA 2011;24:297-304.

3. Bergstrom J: Percutaneous needle biopsy of skeletal muscle in physiological and clinical research. Scand J Clin Lab Invest 1975; 35:609-616.

4. Kristensen JM, Johnsen AB, Birk JB, Nielsen JN, Jensen BR, Hellsten Y, Richter EA, Wojtaszewski JF: Absence of humoral mediated 5'AMP-activated protein kinase activation in human skeletal muscle and adipose tissue during exercise. J Physiol 2007; 585:897-909.

5. Jensen J, Tantiwong P, Stuenaes JT, Molina-Carrion M, DeFronzo RA, Sakamoto K, Musi N: Effect of acute exercise on glycogen synthase in muscle from obese and diabetic subjects. Am J Physiol Endocrinol Metab 2012; 303:E82-89.

6. Lowry OH, Passonneau JV: A collection of metabolite assays. A Flexible System of Enzymatic Analysis 1972:147-218.
