# Supplementary material for: Combined intervention with pioglitazone and n-3 fatty acids in metformin-treated type 2 diabetic patients: improvement of lipid metabolism
Source: Nutr Metab (Lond). 2015 Dec 2;12:52. doi: 10.1186/s12986-015-0047-9 (PMC4667423; doi:10.1186/s12986-015-0047-9)
Supplement: Additional file 2: — The content of linoleic acid (LA) in serum phospholipids. (DOCX 94 kb) [file 12986_2015_47_MOESM2_ESM.docx]

**Additional file 2.** **The content of linoleic acid (LA) in serum phospholipids.**

The analysis was performed using shotgun lipidomics and mass spectrometry data (see Materials and Methods and Fig. 2). Data are mean ± SD in different subgroups at baseline (white bars) and at week 24 (black bars). No significant differences between the subgroups were found.
