# Supplementary material for: Combined intervention with pioglitazone and n-3 fatty acids in metformin-treated type 2 diabetic patients: improvement of lipid metabolism
Source: Nutr Metab (Lond). 2015 Dec 2;12:52. doi: 10.1186/s12986-015-0047-9 (PMC4667423; doi:10.1186/s12986-015-0047-9)
Supplement: Additional file 3: — Serum levels of inflammatory markers in the postprandial state. (DOCX 39 kb) [file 12986_2015_47_MOESM3_ESM.docx]

**Additional file 3. Serum levels of inflammatory markers in the postprandial state.**

The significance threshold for adjusted *p*-values using the Holm-Bonferroni correction is 0.05.

|  | Placebo | Pio | Omega-3 | Pio&Omega-3 |
| --- | --- | --- | --- | --- |
| IL-1RA (pg/ml) |  |  |  |  |
| Baseline | 699 (592, 1029) | 742 (485, 895) | 925 (757, 1424) | 742 (617, 999) |
| Week 24 | 655 (546, 944) | 633 (574, 852) | 919 (642, 1800) | 662 (626, 1056) |
| ∆ | -19 (-87, 7) | 15 (-32, 67) | 23 (-35, 98) | -7 (-163, 53) |
| ∆ (%) | -2.9 (-11.3, 0.5) | 3.0 (-3.8, 7.6) | 2.1 (-4.7, 7.9) | -0.9 (-15.9, 7.2) |
| IL-10 (pg/ml) |  |  |  |  |
| Baseline | 0.39 (0.29, 0.48) | 0.38 (0.33, 0.50) | 0.51 (0.37, 0.65) | 0.34 (0.29, 50) |
| Week 24 | 0.34 (0.27, 0.46) | 0.34 (0.29, 0.40) | 0.40 (0.27, 0.62) | 0.39 (0.27, 0.43) |
| ∆ | -0.02 (-0.05, 0.05) | -0.05 (-0.14, 0.00) | -0.09 (-0.11, -0.05) | 0.00 (-0.05, 0.04) |
| ∆ (%) | -6.9 (-18.9, 17.2) | -14.7 (-26.2, 0.0) | -16.1 (-29.2, -7.3) | 0.0 (-8.1, 9.3) |
| MCP-1 (pg/ml) |  |  |  |  |
| Baseline | 241 (218, 280) | 241 (188, 293) | 256 (191, 312) | 263 (213, 390) |
| Week 24 | 273 (263, 296) | 233 (204, 315) | 256 (194, 307) | 273 (224, 402) |
| ∆ | 22 (-3, 45) | 17 (-22, 36) | 2 (-19, 18) | 15 (-17, 20) |
| ∆ (%) | 10.6 (-1.8, 20.5) | 7.3 (-9.9, 19.0) | 1.4 (-6.0, 6.9) | 6.0 (-4.2, 10.3) |
| CRP (ng/ml) |  |  |  |  |
| Baseline | 1318 (940, 3675) | 1877 (576, 3592) | 2707 (1112, 4133) | 3228 (1403, 5089) |
| Week 24 | 1091 (703, 3494) | 1662 (719, 4369) | 2528 (1089, 4137) | 2739 (1236, 4930) |
| ∆ | -134 (-259, 4) | 114 (-40, 710) | -108 (-203, 88) | -77 (-351, 369) |
| ∆ (%) | -10.8 (-18.8, 0.6) | 8.5 (-10.6, 20.9) | -6.6 (-14.0, 3.0) | -3.3 (-15.1, 0.4) |
| TNF-α (pg/ml) |  |  |  |  |
| Baseline | 8.50 (5.13, 9.16) | 8.21 (6.06, 9.70) | 8.52 (6.85, 10.75) | 8.53 (6.90, 9.95) |
| Week 24 | 8.21 (4.19, 9.10) | 8.37 (5.44, 9.47) | 8.20 (7.06, 9.36) | 8.13 (7.47, 8.89) |
| ∆ | -0.48 (-0.95, 0.30) | 0.00 (-0.62, 0.15) | -0.76 (-1.17, 0.20) | -0.60 (-0.75, 0.04) |
| ∆ (%) | -6.9 (-14.9, 3.4) | 0.0 (-10.2, 1.4) | -5.7 (-13.0, 3.0) | -6.3 (-8.6, -0.4) |
| IL-6 (pg/ml) |  |  |  |  |
| Baseline | 1.55 (0.87, 2.37) | 1.75 (0.98, 2.22) | 1.65 (1.39, 2.15) | 1.40 (1.08, 2.33) |
| Week 24 | 1.37 (1.19, 2.05) | 1.84 (1.11, 2.15) | 2.63 (1.63, 3.05) | 1.48 (1.00, 2.18) |
| ∆ | -0.13 (-0.34, 0.11) | 0.08 (-0.28, 0.43) | 0.00 (-0.20, 1.05) | 0.00 (-0.15, 0.18) |
| ∆ (%) | -5.3 (-16.1, 15.1) | 6.3 (-12.8, 38.3) | 0.0 (-9.8, 66.6) | 0.0 (-12.5, 32.2) |
| sVCAM-1 (ng/ml) |  |  |  |  |
| Baseline | 634 (524, 694) | 522 (476, 670) | 671 (515, 785) | 551 (482, 624) |
| Week 24 | 609 (493, 666) | 541 (470, 613) | 588 (476, 800) | 523 (453, 591) |
| ∆ | -30 (-47, 11) | -10 (-40, 7) | -23 (-64, 30) | -45 (-85, -15) |
| ∆ (%) | -4.6 (-8.7, 1.6) | -1.6 (-6.1, 1.6) | -4.3 (-9.1, 4.9) | -7.3 (-12.1, -2.9) |
| sICAM-1 (ng/ml) |  |  |  |  |
| Baseline | 290 (243, 318) | 301 (243, 352) | 299 (235, 382) | 302 (269, 337) |
| Week 24 | 281 (239, 308) | 274 (223, 336) | 290 (225, 358) | 289 (269, 297) |
| ∆ | -8 (-13, -4) | -11 (-25, -2) | 2 (-23, 15) | -12 (-26, -2) |
| ∆ (%) | -3.1 (-4.5, -1.7) | -3.2 (-8.7, -1.4) | 0.5 (-8.2, 4.4) | -4.9 (-8.8, -0.5) |
| sE-selectin (ng/ml) |  |  |  |  |
| Baseline | 53 (41, 81) | 44 (39, 55) | 42 (36, 65) | 50 (45, 61) |
| Week 24 | 56 (40, 66) | 41 (35, 48) | 42 (34, 63) | 48 (38, 57) |
| ∆ | -3 (-8, -1) | -4 (-5, 0) | 0 (-4, 1) | -4 (-6, -1) |
| ∆ (%) | -5.5 (-13.4, -0.9) | -7.6 (-12.8, -1.0) | -1.1 (--8.1, 0.9) | -7.3 (-13.4, -3.5) |
| sP-selectin (ng/ml) |  |  |  |  |
| Baseline | 76 (63, 86) | 76 (72, 84) | 84 (73, 98) | 83 (65, 93) |
| Week 24 | 69 (65, 88) | 76 (65, 86) | 84 (68, 101) | 71 (60, 85) |
| ∆ | -4 (-6, -1) | -6 (-8, -1) | -4 (-7, 5) | -9 (-12, -6)^a,b^ |
| ∆ (%) | -4.7 (-7.6, -0.9) | -7.7 (-10.8, -1.0) | -5.4 (-10.2, 4.8) | -11.6 (-17.2, -9.1)^a,b^ |
| sPECAM-1 (ng/ml) |  |  |  |  |
| Baseline | 44 (38, 52) | 41 (34, 44) | 39 (33, 46) | 48 (40, 55) |
| Week 24 | 42 (37, 52) | 41 (37, 45) | 38 (32, 46) | 45 (37, 53) |
| ∆ | -3 (-5, 1) | -1 (-3, 4) | -1 (-3, 3) | -2 (-6, 0) |
| ∆ (%) | -7.0 (-10.2, 0.8) | -4.3 (-5.7, 12.6) | -3.8 (-10.2, 8.4) | -4.9 (-14.9, -0.1) |
| sCD105 (ng/ml) |  |  |  |  |
| Baseline | 6.47 (5.22, 7.42) | 5.77 (4.29, 7.22) | 6.74 (4.15,7.82) | 5.64 (4.21, 6.43) |
| Week 24 | 6.37 (4.86, 7.45) | 6.17 (4.37, 7.29) | 5.92 (4.37, 7.76) | 5.24 (4.28, 6.14) |
| ∆ | 0.00 (-0.90, 0.75) | -0.53 (-0.93, 0.40) | -0.23 (-1.31, 0.16) | 0.00 (-0.74, 0.43) |
| ∆ (%) | 0.0 (-13.6, 19.9) | -6.4 (-15.2, 8.2) | -3.0 (-19.0, 6.4) | 0.7 (-11.8, 8.1) |

Data are a median and interquartile range (Q1, Q3). Levels of various cytokines in serum samples collected at 120 min of the meal test (see Figure 4), at baseline and at week 24 were determined by microbead Luminex® assay (Luminex Corporation, Texas, United States), following product protocol provided by R&D Systems Inc. (Minneapolis, United States). The samples were analysed using Bio-Plex® 200 (Bio-Rad Laboratories, California, United States). The analytes were identified by bead specific laser and quantified by the magnitude of streptavidin-phycoerythrin signal in comparison to standard signals used to produce a standard curve using Bio-Plex Manager software version 6.1 (Bio-Rad Laboratories, California, Unite States).. Anti-inflammatory cytokines: IL-1RA (interleukin-1 receptor antagonist ), IL-10 (interleukin-10). Pro-inflammatory cytokines: MCP-1 (monocyte chemoattractant protein-1), CRP (C-reactive protein), TNF-α (tumor necrosis factor- α), IL-6 (interleukin-6). Cell adhesion molecules: sVCAM-1 (soluble vascular cell adhesion molecule-1), sICAM-1 (soluble intercellular adhesion molecule-1), sE-selectin (soluble endothelial adhesion molecule 1), sP-selectin (granule membrane protein 140), sPECAM-1 (soluble platelet-endothelial cell adhesion molecule-1). Neovascularization marker: sCD105 (endoglin). Serum inflammatory markers were ∆, a difference between week 24 and baseline values. ∆ (%) a difference between week 24 and baseline values in % of the baseline value.  ^a,b^Significant differences (Kruskal-Wallis test) compared with Placebo and Omega-3, respectively.
