# Supplementary material for: Combined intervention with pioglitazone and n-3 fatty acids in metformin-treated type 2 diabetic patients: improvement of lipid metabolism
Source: Nutr Metab (Lond). 2015 Dec 2;12:52. doi: 10.1186/s12986-015-0047-9 (PMC4667423; doi:10.1186/s12986-015-0047-9)
Supplement: Additional file 4: — Correlations between changes in Omega-3 PhL Index and changes in selected variables in response to interventions. (DOCX 22909 kb) [file 12986_2015_47_MOESM4_ESM.docx]

**Additional file 4.** **Correlations between changes in Omega-3 PhL Index and changes in selected variables in response to interventions.**

1. Correlation between changes in Omega-3 PhL Index (∆ Omega-3 PhL Index)

and in HbA_1c_ serum levels (∆ HbA_1c_)

**
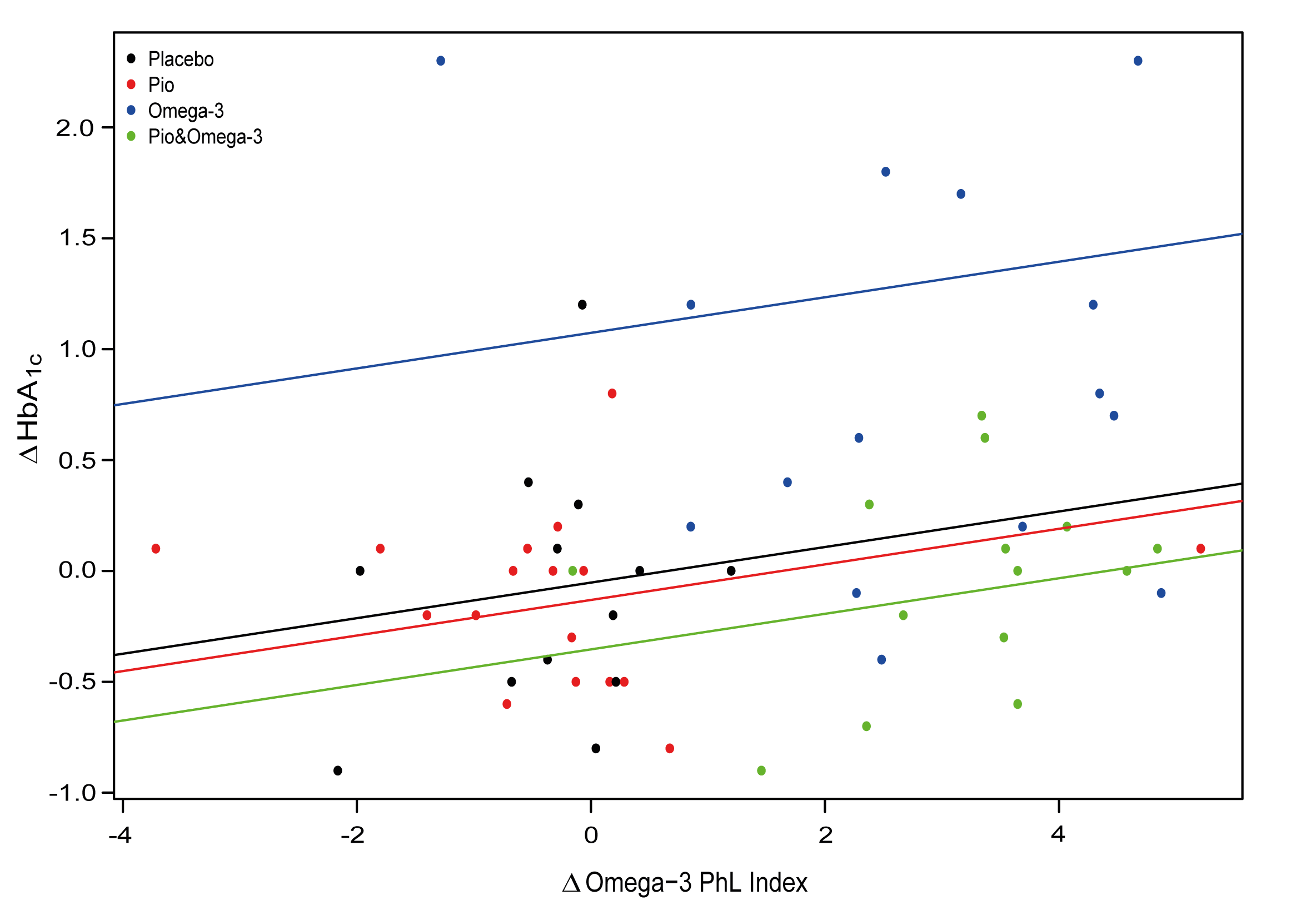
**

1. Correlation between changes in Omega-3 PhL Index (∆ Omega-3 PhL Index)

and in AUC NEFA serum levels (∆ AUC NEFA)

**
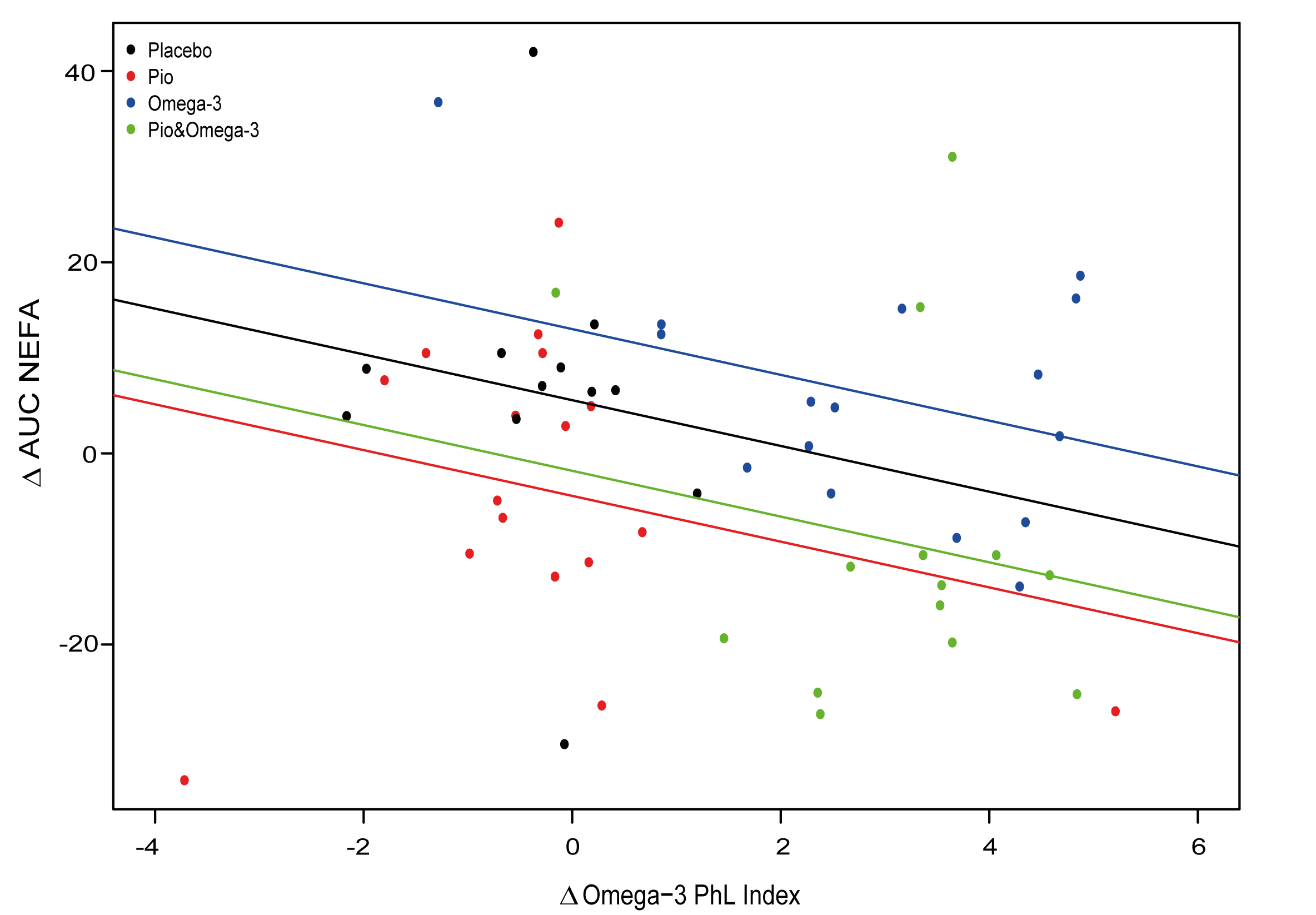
**

Analysis was performed using Pearson correlation using the data shown in Table 1 (Omega-3 PhL Index), Table 2 (HbA_1c_) and Figure 4 (AUC NEFA). For both correlations, *p* < 0.1. ∆, a difference between week 24 and baseline values.
